# Narrativas de adoecimento e alternativas textuais: o câncer de mama em quadrinhos

**DOI:** 10.1590/0102-311XPT049323

**Published:** 2023-06-26

**Authors:** Waleska de Araújo Aureliano

**Affiliations:** 1 Instituto de Ciências Sociais, Universidade do Estado do Rio de Janeiro, Rio de Janeiro, Brasil.

As narrativas sobre processos de adoecimento crônico revelaram-se um campo de análise importante para produção de conhecimentos sobre esses fenômenos, que vai além de sua dimensão estritamente biológica. Na Antropologia, as narrativas produzidas por adoecidos e outros agentes com eles envolvidos, como profissionais de saúde, familiares e comunidade, tornaram-se objeto de investigação para muitas/os pesquisadoras/res ^1^^,^^2^^,^^3^. Embora narrar os estados de aflição e sofrimento não seja a única maneira de retratar a vivência com uma enfermidade, esse seria o meio primário para dar forma a essa experiência e torná-la disponível não só para os outros, mas para o próprio sujeito que narra. Por meio das construções narrativas seria possível organizar sentidos e saberes em torno desse evento que, quase sempre, provoca deslocamentos da percepção corporal e demanda do sujeito novas e reiteradas formas de agência nas suas relações com o mundo e com os outros.

Nesse sentido, o livro *180º: Minhas Reviravoltas com o Câncer de Mama*^4^ nos apresenta uma proposta de narrativa em primeira pessoa na qual se evidenciam constantemente o caráter relacional dos processos de adoecimento e a multiplicidade de atores sociais envolvidos no seu enfrentamento. A história de Carolina Passos, personagem que representa a autora Dulce Ferraz ^(^mas que pode representar inúmeras mulheres^)^, é desenvolvida em um formato original, ainda pouco utilizado no Brasil quando se trata da literatura sobre saúde: as histórias em quadrinho ^(^HQs^)^, em sua forma estendida, as *graphic novels*. Diagnosticada com câncer de mama aos 36 anos, a autora conta na apresentação do livro como foi estimulada por outras mulheres, entre elas a antropóloga Soraya Fleischer da Universidade de Brasília, a registrar essa experiência por meio da escrita. Desses registros nasceu a ideia do livro, empreitada dividida com Fleischer, com a também antropóloga Fabiene Gama, da Universidade Federal do Rio Grande do Sul, e com a grafiteira e designer Camila Siren, autora das belíssimas ilustrações da obra. Essa relação de amizade e trabalho, permeada pela influência da Antropologia, contribuiu para colocar em diálogo as perspectivas do cuidado, a abordagem autoetnográfica e a Medicina Gráfica, resultando em uma obra inovadora dentro do vasto universo de relatos pessoais sobre o câncer de mama, que emergiram a partir dos anos 1980 e se produzem com bastante intensidade na contemporaneidade ^5^^,^^6^.

A *graphic novel* está dividida em três capítulos de leitura fluída e leve, apesar do tema denso e dos termos técnicos que, por vezes, fazem parte do enredo. Além dos quadrinhos, o livro contém textos complementares que abordam desde o trabalho de concepção e produção da obra até como ela poderá ser utilizada por diferentes pessoas, de pacientes a gestores em saúde. Acompanhamos a personagem principal em três momentos distintos: ^(^1^)^ a busca pelo diagnóstico; ^(^2^)^ a definição dos tratamentos, fase permeada por muitas negociações ^(^por vezes tensas e conflituosas^)^ com profissionais de saúde e apoiada na escuta de outras mulheres que passaram pela doença; e ^(^3^)^ a realização dos tratamentos, quando a vida passa girar não em torno da doença, mas com a doença, trazendo dores, limitações e perdas, mas também aprendizados e construção de projetos, como o próprio livro.

O fato de a personagem/autora ser uma pesquisadora em saúde coletiva é central para a construção da narrativa, uma vez que esse pertencimento lhe deu, segundo ela, ferramentas técnicas e teóricas para interpretar sua experiência. No entanto, isso não quer dizer que o único saber legitimado deve ser o da ciência. Ao mesmo tempo que busca pesquisas científicas e diferentes opiniões médicas sobre seu tratamento, nossa personagem se envolve em redes de compartilhamento de informações formadas por outras mulheres que viveram ou estão vivendo um câncer. Conversar com quem já trilhou esse caminho é apresentado como algo fundamental para o enfrentamento da doença, uma vez que coloca em diálogo saberes técnicos e práticos.

Como muitas mulheres, Carolina/Dulce sofre o impacto inicial do diagnóstico, o medo da morte ^(^“*quero ver minhas filhas crescerem*”^)^ e dos efeitos colaterais dos tratamentos inicialmente indicados, entre eles 16 sessões de quimioterapia. Sua postura questionadora e reflexiva muda radicalmente essa prescrição. O acesso a uma medicina de precisão, ausente no sistema público de saúde e mesmo nos convênios privados, a livra de uma quimioterapia desnecessária - e a autora tem consciência do privilégio de sua condição. Nesse sentido, critica o termo “sobrevivente do câncer”, pois reconhece que essa sobrevivência nada tem a ver com uma luta individual, ela é fruto de dinâmicas sociais como rede de apoio, cuidado de terceiros, acesso adequado a tratamentos e tecnologias em saúde.

Dois textos curtos que também compõem o livro apresentam alternativas textuais e teóricas para se pensar a popularização da informação médica e científica e a construção de conhecimentos e cuidados em saúde. Explorando as relações entre arte, literatura e saúde, Dulce Ferraz expõe dados interessantes sobre como a partir da segunda metade do século XX emerge o campo da Medicina Narrativa, que valoriza as histórias contadas por pessoas doentes e por quem cuida delas, as chamadas patografias. Entre as formas de registro dessas narrativas, as HQs despontaram como uma maneira mais direta, íntima e pessoal de narrar eventos de adoecimento e comunicar essa experiência para família, comunidade e profissionais de saúde ao ponto de tornar-se uma área específica conhecida como Medicina Gráfica. Embora ainda tímida no Brasil, a Medicina Gráfica está em expansão em contextos euro-americanos e já conta com congressos, publicações de dossiês e artigos científicos sobre o tema, além de *sites* voltados para divulgar a crescente produção da área.

Fabiene Gama, por sua vez, nos apresenta a Autoetnografia, proposta metodológica e textual que tem como base a reflexividade da pesquisadora sobre si mesma, tratando de questões de ordem social a partir de uma experiência pessoal, aproximando-se, assim, da máxima do feminismo que diz que “*o pessoal é político*”. No entanto, Gama nos lembra que, apesar de baseada em uma história individual, a Autoetnografia “*é sempre construída de forma dialética, relacional, contextual, levando em consideração experiências de outras pessoas e também as pesquisas já realizadas sobre o tema*” ^(^p. 303^)^. Na literatura sobre temas da saúde, reflexões sociais baseadas em experiências pessoais já se fazem presentes pelo menos desde a década de 1980, sendo o trabalho de Susan Sontag ^7^ talvez um dos mais destacados nesse sentido. Antropólogas fizeram exercício semelhante em artigos, refletindo criticamente sobre determinados aspectos do campo da saúde a partir de suas experiências pessoais de adoecimento ^8^^,^^9^. A contribuição mais recente da Autoetnografia está em situar o texto etnográfico entre arte e ciência, permitindo experimentações textuais e visuais ao buscar formas mais atraentes de apresentar as reflexões suscitadas, algo que *180º: Minhas Reviravoltas com o Câncer de Mama* realiza com maestria ao apostar no formato de HQs.

Por fim, um detalhe importante a mencionar são as escolhas feitas para representação imagética das personagens. Como forma de questionar estereótipos presentes no campo na saúde, em que médicos e especialistas são concebidos quase sempre como homens brancos, vemos nessa *graphic novel* médicos negros, cientistas mulheres, corpos com diferentes tons de pele e em papéis sociais variados. Essa é mais uma qualidade das narrativas, que aqui é explorada estética e politicamente: possibilitar a construção social da realidade, e não meramente representá-la.
